# Acquired isolated factor VII deficiency in a patient with myxoid pleomorphic liposarcoma, case report

**DOI:** 10.1097/MD.0000000000036621

**Published:** 2023-12-29

**Authors:** Mansour Aljabry, Manar Aleid, Shahad Almutairi, Reema AlSerhani, Shahad Alsahil, Ghazi Alotaibi

**Affiliations:** a Department of Pathology, College of Medicine, King Saud University, Riyadh, Saudi Arabia; b Department of Medicine, King Fahad Medical City, Riyadh, Saudi Arabia; c College of Medicine, King Saud University, Riyadh, Saudi Arabia; d Hematology/Oncology Division, Department of Medicine, College of Medicine, King Saud University, Riyadh, Saudi Arabia.

## Abstract

**Introduction:**

Acquired factor VII (FVII) deficiency is a rare condition with various causes, including acquired inhibitors to FVII, liver disease, and malignancies. Myxoid pleomorphic liposarcoma is a rare and aggressive form of soft tissue sarcoma that can cause a range of clinical manifestations, including bleeding and clotting disorders.

**Patient concerns and diagnosis:**

We present a case report of a 21-year-old man with severe acquired FVII deficiency due to mediastinal myxoid pleomorphic liposarcoma. The patient presented with elevated International normalized ratio (INR) and a severe reduction in FVII coagulant activity, unresponsive to conventional therapy. While an acquired inhibitor to FVII was initially suspected, negative results from laboratory testing, including protein G sepharose adsorption and a Bethesda assay using Immunoglobulin G purified from patient plasma, made the diagnosis of an acquired inhibitor to FVII uncertain.

**Interventions and outcome:**

The patient underwent surgical resection of the tumor, supported by recombinant FVII infusion, leading to the normalization of coagulation parameters. However, a relapse of the disease was detected 6 months later when he was noted to have a decline in FVII levels.

**Conclusion:**

This case highlights the importance of considering rare causes of bleeding and clotting disorders, particularly in unresponsive or atypical presentations. It also underscores the need for close monitoring and follow-up in patients with acquired FVII deficiency, even after successful treatment.

## 1. Introduction

Factor VII (FVII) plays an important role in the initiation of blood coagulation, forming a complex with tissue factor, which activates FIX and FX, and FVII zymogen.^[1]^ FVII deficiency can be caused by genetic mutations that affect the synthesis or function of FVII or by acquired conditions that affect the liver, where FVII is produced. The severity of the bleeding disorder can vary widely, ranging from mild bleeding tendencies to life-threatening hemorrhages.^[2]^

The presence of an acquired deficiency to factor VII is a rare but potentially serious complication. This inhibitor is an antibody that targets FVII and prevents its activation, leading to a prolonged bleeding time. In some cases, the reduction in FVII:C levels can be corrected with fresh frozen plasma or vitamin K administration. Acquired deficiency, not linked to liver disease or vitamin K deficiency, is extremely rare.

Several cases have reported a correlation between factor VII deficiency and conditions such as severe systemic sepsis, malignancies, and stem cell transplantation. Symptoms can vary from no symptoms to severe bleeding that can be life-threatening. Factor VII deficiency should be suspected if a patient has a prolonged prothrombin time that does not correct completely after vitamin K administration.

## 2. Case report

A 21-year-old male nonsmoker, a university student with a significant surgical history of left adrenalectomy during childhood at the age of one for a left adrenal tumor. The patient was following up in another hospital because of a 6-month history of generalized weakness, fatigue, and significant weight loss of 22 kg with poor appetite. The patient also complained having palpitations, dizziness for 1 year, and 1 episode of syncope.

The patient was referred to our tertiary care hospital because of worsening symptoms and developing new complaints; he started having parasternal chest pain in the 5th to 7th intercostal space, left shoulder, and back pain, which was progressive and increased at night and occasional night sweating for 3 months prior to referral. No family history of malignancy or blood disorders was present.

A malignancy workup was carried out in which the Computed tomography (CT) chest showed a large heterogeneous hyper enhancing solid anterior mediastinal mass and bilateral pleural effusion. A true-cut biopsy was taken from the mass, which showed high malignant pleomorphic neoplasm favoring pleomorphic myxoid liposarcoma diagnosis.

He received single-agent doxorubicin as a neoadjuvant to shrink the tumor. Tumor rapture bilateral pleural pigtails were inserted to drain the effusion, and intrapleural tissue Plasminogen Activator was injected into the right side due to loculated effusion. The patient started to bleed massively from the right-sided catheter. He was immediately shifted to the intensive care unit (ICU) as a case of hemorrhagic shock requiring inotropic support and transfusion of 2 units of packed red blood cells (RBCs) as well 6 units of fresh frozen plasma (FFP) along with 1 gm of tranexamic acid. A repeat CT chest showed an enlargement of the previous mediastinal mass with secondary internal bleeding within the tumor.

The patient had a deranged coagulation profile during admission with a prolonged PT of 20 seconds (*R* = 12.1–15.7 seconds) and aPTT of 48 seconds (*R* = 25.7–39.5 seconds). FVII was sent in which it came to be 22% (*R* = 40–170%), and FV and FVIII levels came to be normal. The patient doesn’t have any personal or family history of bleeding.

He received 3 doses of oral vitamin K, each 10 mg which showed improvement in the International Normalized Ratio without normalization. Then 1 dose of recombinant factor VII (NovoSeven) 30 µg/kg was administered immediately before the surgery.

Three months later, patient underwent right sided HemiClamshell and thoracotomy, excision of the mediastinal mass and decortication of the right lung as well asl wedge resection of the middle lobe. The patient was admitted to the ICU post-op with an estimated blood loss of 4 liters. He received 16 units of packed red blood cells and 18 units of FFP. A bilateral chest tube was inserted, which drained 500 mL bilaterally bloody. A bilateral femoral line and left radial arterial lines were maintained.

He then continued receiving NovoSeven for 6 days post-surgery with adequate hemostasis. The chest tube and peripherally inserted central catheter were removed, and the patient was discharged in good condition.

## 3. Outcome and follow-up

The level of FVII was tested 3 days after stopping NovoSeven, and it came to 79%. The repeat PT and APTT levels were normal, and FVII was 100% at 10 days follow-up in the hematology clinic after the discharge.

The patient’s health improved significantly, and a month later, he started receiving adjuvant chemotherapy with AIM protocol s/p ¼ cycles. His FVII was 76% at the 4-month follow-up, and he was generally doing well.

Unfortunately, he was again admitted to the hospital 6 months later, complaining of back ache with right scapula, cervical spine pain, and dyspnea. Upon investigations, he was found to have disease recurrence with metastasis. A palliative care plan was applied in the management of the patient. He received only narcotics during his last admission, according to the plan. After a short period, the patient’s health deteriorated, and he expired due to cardiac arrest.

## 4. Discussion

This case report illustrates the acquired deficiency of FVII secondary to malignancy which could be explained by several possible mechanisms, including decreased synthesis, accelerated consumption or catabolism, neutralization by autoantibodies, synthesis of a qualitatively abnormal hypoactive FVII molecule and abnormal adsorption by tumor cells.^[3,4]^

Only a few cases have been reported in the literature for the acquired FVII deficiency associated with malignancy. Franchini et al^[5]^ published a case of a patient admitted for a lung mass investigation and found to have prolonged PT and aPTT with a low FVII level. Bronchoscopic removal of the mass was done, and only 1 dose of 20 µg/kg of Recombinant FVII was given 30 minutes before the procedure; no bleeding episodes have been reported. The histopathology report confirmed the diagnosis of bronchogenic squamous cell carcinoma. The patient underwent chemoradiotherapy, improving FVII levels.

De Raucourt et al^[6]^ reported a case of pleural liposarcoma who was found to have a prolonged PT, which is not corrected with vitamin K, FVII sent and came to be low. The patient received neoadjuvant chemotherapy, then surgical removal followed by radiation therapy, FVII level was repeated 4 months after the completion of treatment and came to be normal. Later, he experienced a relapse and recurrence of the disease, and his FVII level fell again.

Moosavi et al^[7]^ studied a case of acute myelogenous leukemia with an associated trisomy 8 cytogenetic abnormality with no history of bleeding disorders before and was found to have a low FVII level which normalized after the induction chemotherapy.

Mullighan et al^[8]^ published a case of a 53-year-old male who underwent coronary artery bypass surgery, which was complicated by surgical site infection and bacteremia and was found to have low FVII. He underwent an attempt at reconstructive surgery, which was aborted due to severe bleeding that was not controlled with FFP, vitamin K, and antifibrinolytic therapy. The patient was started on recombinant FVII with good hemostasis. FVII was repeated 3 months after treating the infection and was normal.

As there is a wide spectrum of the manifestation of FVII deficiency among patients and response to the lines of management, Lee et al^[9]^ published a case of a pregnant lady with no previous history of a bleeding disorder, admitted with active labor, investigated for FVII level due to isolated prolonged PT. The level of FVII came to be low. She was delivered by cesarean section because of labor dystocia with a support of 6 units of FFP, tranexamic acid, and IVIG. The procedure went uneventful, with excellent hemostasis.

## 5. Conclusion

Factor VII deficiency should be suspected in patients with isolated prolonged PT that is not entirely corrected after vitamin K administration, even if the patient has no personal or family history of bleeding in the past. Moreover, other vitamin k-dependent factors should be checked, also mixing studies to rule out the possibility of inhibitor presence. Prophylactic recombinant factor VII is advisable in such cases prior to any interventions.

## Author contributions

**Conceptualization:** Mansour Aljabry, Ghazi Alotaibi.

**Data curation:** Mansour Aljabry, Manar Aleid, Reema AlShahrani, Shahad Alsahil, Shahad Almutairi, Ghazi Alotaibi.

**Formal analysis:** Mansour Aljabry, Manar Aleid, Reema AlShahrani, Shahad Almutairi, Ghazi Alotaibi.

**Investigation:** Mansour Aljabry, Shahad Alsahil, Shahad Almutairi.

**Methodology:** Mansour Aljabry, Reema AlShahrani, Shahad Alsahil, Shahad Almutairi, Ghazi Alotaibi.

**Project administration:** Mansour Aljabry.

**Resources:** Manar Aleid.

**Software:** Mansour Aljabry, Ghazi Alotaibi.

**Supervision:** Mansour Aljabry.

**Writing – original draft:** Mansour Aljabry, Manar Aleid, Reema AlShahrani, Shahad Alsahil, Shahad Almutairi, Ghazi Alotaibi.

**Writing – review & editing:** Mansour Aljabry.
